# Intrathecal Anti-*Akkermansia muciniphila* IgG Responses in Multiple Sclerosis Patients Linked to CSF Immune Cells and Disease Activity

**DOI:** 10.3390/jcm14165771

**Published:** 2025-08-15

**Authors:** Carolina Cruciani, Camille Mathé, Marco Puthenparampil, Paula Tomas-Ojer, Maria José Docampo, Roland Opfer, Ilijas Jelcic, Arnaud B. Nicot, David-Axel Laplaud, Roland Martin, Mireia Sospedra, Laureline Berthelot

**Affiliations:** 1 Neuroimmunology and MS Research (NIMS), Department of Neurology, University Hospital Zurich, Frauenklinikstrasse 26, 8091 Zürich, Switzerlandmarco.puthenparampil@unipd.it (M.P.); mariajose.docampo@uab.cat (M.J.D.);; 2 Centre de Recherche Translationnelle en Transplantation et Immunologie, CR2TI UMR1064, Inserm, Université de Nantes, CHU de Nantes, CEDEX 01, 44093 Nantes, France; 3 Department of Neuroscience DNS, University Hospital of Padova, Via Giustiniani, 5, 35128 Padova, Italy; 4 Jung Diagnostics GmbH, HIP—Health Innovation Port, Röntgenstraße 24, 22335 Hamburg, Germany; roland.opfer@jung-diagnostics.de

**Keywords:** multiple sclerosis, gut microbiota, dysbiosis, *Akkermansia muciniphila*, antibodies, cerebrospinal fluid

## Abstract

**Background/Objectives**: Gut microbial dysbiosis, leaky gut, and increased transepithelial translocation of commensal bacteria have been documented in multiple sclerosis (MS). Intrathecal IgGs specific for *Akkermansia muciniphila*, a gut bacterium, are increased in patients with MS and associated with clinical disability. Our objective here was to explore the putative involvement of intrathecal anti-*A. muciniphila* IgG in MS pathogenesis by characterizing patients with different anti-*A. muciniphila* IgG indices. **Methods**: Serum and intrathecal IgG specific for *A. muciniphila* and other gut bacteria, as well as routine cerebrospinal fluid (CSF) parameters, were measured in 61 patients with MS. Examination of these patients included immunophenotyping of CSF-infiltrating and paired circulating lymphocytes, intrathecal markers of neurodegeneration and inflammation, and a detailed characterization of demographic, clinical, and magnetic resonance imaging (MRI) features. **Results**: Plasma blasts (*p* < 0.01), B cells (*p* < 0.01), and Th2 cells (*p* < 0.01), which might be involved in antibody production, were increased in the CSF of these patients, as well as blood pro-inflammatory Th17 cells (*p* < 0.05). Anti-*A. muciniphila* IgG indices were negatively associated with blood-brain barrier (BBB) permeability and circulating monocytes (*p* < 0.001), and positively with brain lesion load (*p* < 0.01). **Conclusions**: The differences between patients with low and high anti-*A. muciniphila* IgG indexes regarding BBB permeability, CSF cell infiltrates, and pro-inflammatory peripheral immune cells, as well as imaging features, support a role of anti-*A. muciniphila* immune response in MS pathogenesis.

## 1. Introduction

MS is an immune-mediated demyelinating disease of the central nervous system (CNS) [1,2] that develops in genetically susceptible individuals and likely requires environmental triggers [3,4]. Recently, several studies have revealed a dysbiosis in the gut microbiota of MS patients, which might play a role in disease pathogenesis [5]. Supporting this hypothesis, it has been demonstrated that the transfer of gut microbiota from patients with MS, but not from healthy controls, into mice can induce or exacerbate experimental autoimmune encephalomyelitis [6,7]. Since gut microbiota and their metabolites are important for maintaining the gut epithelial barrier [8] and influence systemic immunity [9,10], microbiota dysbiosis affects the intestinal barrier function and immune homeostasis. In addition, gut enrichment in mucin-degrading bacteria can reduce mucus thickness and facilitate mucosal damage [11]. A leaky gut can then enable the translocation of commensal bacteria across the intestinal epithelium [12], enabling activation of the immune system. Bacterial lipopolysaccharides (LPSs) and metabolites from translocated bacteria can access the blood and circulate to distant organs and affect BBB permeability, leading to brain infiltration [13], as well as the maturation and function of microglia [14] and astrocytes [15]. Supporting the role of leaky gut in MS pathogenesis, altered biomarkers of gut barrier leakiness are common in patients with MS and correlate with disease progression and with increased BBB permeability [16,17]. Furthermore, circulating LPS, as a measure of gut microbiota translocation, is increased in MS patients and correlates with disability [18].

Among the different bacteria reported to be enriched in patients with MS [6,7,19], one of the most interesting is the mucin-degrading *Akkermansia muciniphila* [11]. We recently demonstrated higher intrathecal levels of anti-*A. muciniphila* immunoglobulin G (IgG) in patients with MS compared with controls and a significant correlation with disease disability [20], which supports a putative role of this bacterium in MS. Furthermore, two studies demonstrated cross-recognition between recently identified MS autoantigens and *A. muciniphila*-derived peptides by CD4+ T cells from patients with MS [21,22].

In order to better understand the involvement of intrathecal anti-*A. muciniphila* IgG in MS, we identified patients with MS with different anti-*A. muciniphila* IgG indices and characterized them in depth by analysis of CSF measures, ex vivo immunophenotyping of CSF, and paired blood samples, as well as demographic, clinical, and MRI characteristics.

## 2. Materials and Methods

### 2.1. Patient Material

Paired CSF and blood samples were collected from 61 untreated patients with MS (Table 1). This study was approved by the Contonal Ethics Committee of Zurich (EC-No. 2013-0001 approved on 5 June 2013). Informed consent was obtained from all patients. All CSF samples were obtained for diagnostic purposes. Blood samples were collected on the same day. Patients were recruited from the Neuroimmunology and MS Research Section, Neurology Clinic, University Hospital, Zurich. Diagnosis was based on the revised McDonald criteria [23]. Fifty patients had never been treated. Eleven had previously been treated but were considered untreated at the time of lumbar puncture (seven patients received steroids, not during the last 4 weeks prior to enrolment, and four patients received glatimer acetate, not during the last 3 months prior to enrolment). Twenty-one patients were excluded from the analysis as we took the top twenty low patients (anti-*A. muciniphila* IgG index from 0.005 to 0.0043) and the top twenty high-index patients (from 0.063 to 0.24).

### 2.2. Quantification of Anti-Bacterial Antibodies

ELISA tests were performed on paired unfrozen serum and CSF samples from −80 °C storage, as previously described, to detect antibodies against *Akkermansia muciniphila* [20]. Bacterial proteins were coated at 1 µg/mL in phosphate buffer saline (PBS) overnight at 4 °C. Blocking was performed using Bovine serum albumin (Sigma Aldrich, Paris, France) at 1% in PBS for 1 h at 37 °C. Patient samples were incubated for 2 h at 37 °C in PBS with 1% bovine serum albumin (dilutions 1/100 for serum, 1/10 for CSF). Anti-human IgG antibodies coupled with horseradish peroxidase (Bethyl Laboratories, Nanterre, France) at 1/5000, incubated for 1 h at 37 °C, were used for detection. The reaction with the substrate (3,3′,5,5′-Tetramethylbenzidine, BD Biosciences, Le Pont de Claix, France) was stopped with sulfuric acid (0.18 M, Sigma Aldrich) after 10 min. Plates were read at 450 nm using a Spark 10M multimode microplate reader (Tecan, Grodig, Austria). This ELISA test with ROC analysis showed that the AUC was 0.78 (95% CI = 0.065–0.90) with a *p* < 0.0058 for the dosage of anti-*Akkermansia muciniphila* IgG in CSF comparing patients with MS with non-inflammatory neurological disease patients. Sensitivity was 61.9, and specificity was 84.21.

### 2.3. Routine CSF and Serum/Blood Measures

CSF measures were determined as previously reported [24]. CSF-infiltrating cells were counted with a Fuchs–Rosenthal counting chamber under the microscope within 1 h after lumbar puncture. CSF total protein, as well as CSF and serum albumin, immunoglobulin (Ig) G, IgM, and IgA, were determined by immunonephelometry. Intrathecal indices for anti-*A. muciniphila* IgG were calculated using the following formula (Ig Index = (Ig CSF/Alb CSF)/(Ig Serum/Alb Serum)). Quotients (Q) were defined as Q = (concentration in CSF [mg/L]/concentration in serum [g/L]). Because QAlb increases with age, we calculated a maximum normal QAlb (QNorm) for each patient, which takes into account the age at lumbar puncture (QNorm = [age/15] + 4 × 10^−3^). Intrathecal neurofilament light chain (NF-L) and chitinase 3-like 1 (CHI3L1) proteins were quantified in CSF samples by ELISA (Uman Diagnostics, Umea, Sweden, and MicroVue, Athens, OH, USA, respectively) according to the manufacturer’s instructions. Routine blood analyses were performed in the Hematology Department, USZ. Total white cells were counted with an automated counter. The number of neutrophils, basophils, eosinophils, and monocytes was manually determined using Romanofsky staining.

### 2.4. HLA Typing

Patients were typed for HLA-class II (DRB1*, DRB3*, DRB4*, DRB5*, DQA1*, and DQB1*) molecules using high-resolution HLA sequence-based typing at Histogenetics LLC, Ossining, NY, USA. Isolation of DNA from whole blood with a final concentration of 15 ng/μL was performed with a standard DNA isolation protocol using a Triton lysis buffer and proteinase K treatment.

### 2.5. Immunophenotyping

**Figure 1:**
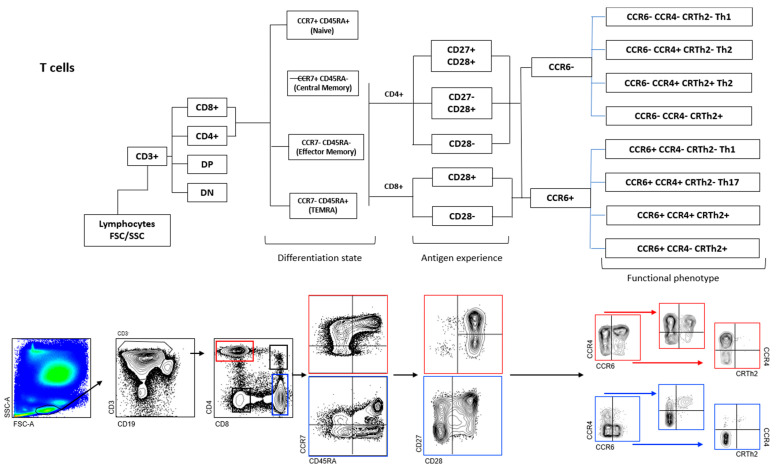

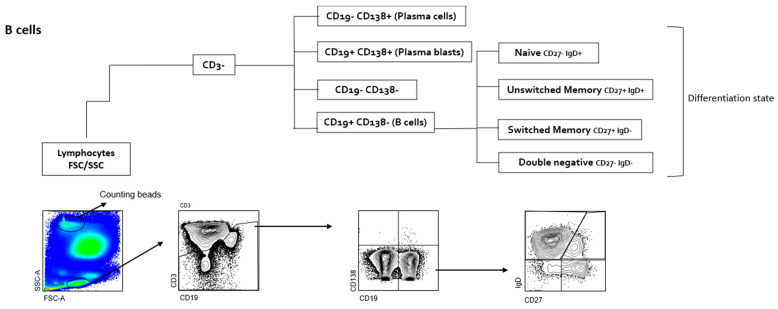
Gating strategy of T and B cells. Doublets were excluded, followed by identification of lymphocytes by size. Next, CD3− are identified and among them, plasma cells (CD19− CD138+), plasma blasts (CD19+ CD138+), B cells (CD19+ CD138−) and CD19− CD138− cells. Among B cells, naïve (IgD+ CD27−), unswitched memory (IgD+ CD27+), switched memory (IgD− CD27+), and doublé negative (IgD− CD27−) B cell subsets are also identified. In CD3+ T cells, CD3+ CD4+ and CD3+ CD8+ cells are first identified and then separated in CM (CCR7+ CD45RA−), EM (CCR7− CD45RA−), TEMRA (CCR7− CD45RA+) and naive (CCR7+ CD45RA+). CM, EM, and TEMRA CD8+ T cells are then separated into CD28+ and CD28−, while CM, EM, and TEMRA CD4+ T cells are separated into CD28+ CD27+, CD28+ CD27−, and CD28−. Each one of these CD4+ and CD8+ T cells are separated first in CCR6− and CCR6+ and then in Th1 (CCR6− CCR4− CRTH2−), Th2-A (CCR6− CCR4+ CRTH2−), Th2-B (CCR6− CCR4+ CRTH2+), CCR6− CCR4− CRTH2+, Th1* (CCR6+ CCR4− CRTH2−), Th17 (CCR6− CCR4+ CRTH2−), CCR6+ CCR4+ CRTH2+ and CCR6+ CCR4− CRTH2+ cells.

Flow cytometric immunophenotyping of CSF-infiltrating and paired circulating lymphocytes was performed as previously reported on fresh samples [24]. Antibodies: anti-CD3 AF700, anti-CD4 PE TR, anti-CD8 BV510, anti-CD45RA BV711, anti-CCR7 BV421, anti-CD27 APC Cy7, anti-CD28 PE Cy7, anti-CCR4 APC, anti-CRTh2 PE, anti-CCR6 BV785, anti-CD19 PerCPCy5.5, anti-IgD BV605 and anti-CD138 FITC. SPHEROTM AccuCount Particles (Sperotech, Inc., Lake Forest, IL, USA) were added to determine absolute counts following the manufacturer’s instructions. Sample acquisition was performed in an LSR Fortessa cytometer (BD Biosciences, Franklin Lakes, NJ, USA), and data were analyzed using FACSDiva (version 6.0, BD Biosciences) and FlowJO (version 10.0, TreeStar Inc., Ashland, OR, USA) software. The gating strategy is summarized in Figure 1.

### 2.6. Magnetic Resonance Imaging

Patients were scanned with a 3T Philips Ingenia or 3T Siemens Skyra. The MRI protocol included a 3D fluid-attenuated inversion recovery (FLAIR) sequence. The number and the total volume in ml of all hyperintense lesions were determined from the FLAIR images by an automatic algorithm based on convolutional neural networks [25]. Whole brain volume in ml was determined on the pre-contrast MPRAGE image using the automatic processing pipeline Biometrica MS^®^ analysis platform (version 2.1, jung diagnostics GmbH, Hamburg, Germany).

### 2.7. Statistics

Statistical analysis was performed using GraphPad Prism 8.0 (GraphPad Software, La Jolla, CA, USA). For the comparison of two groups of patients, we used the U-test (Mann–Whitney) for non-normally distributed variables. For the comparison of more than two groups of patients, we used Kruskal–Wallis test for non-normally distributed variables. Linear correlation between variables was tested using Spearman’s r for not-normally distributed variables. The significance level was set at *p* < 0.05. Associations were calculated using Fisher’s Exact Test with a 0.05 significance.

## 3. Results

### 3.1. IgGs Specific for Gut Commensal Bacteria

**Figure 2:**
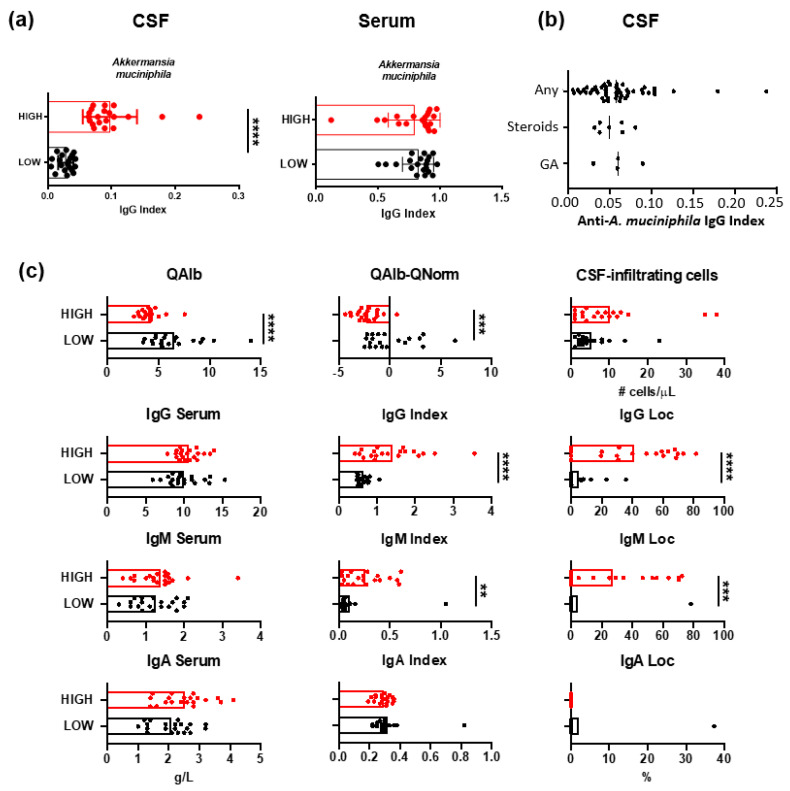
IgG specific for *A. muciniphila* bacteria in MS and their association with CSF measures. (**a**) IgG index and serum levels of IgG specific for *A. muciniphila* in patients with MS. (**b**) Anti-*A. muciniphila* IgG indices in patients with MS never treated, previously treated with steroids, and previously treated with glatiramer acetate (GA). (**c**) CSF measures in patients with high (red) and low (black) anti-*A. muciniphila* IgG indices. Each dot in the graphs corresponds to a single patient, and the bars show the mean. The Kruskal–Wallis test was used to compare more than two groups of patients, and the Mann–Whitney test to compare two groups. Linear correlation between variables was tested using Spearman’s r correlation. Statistical significance (** *p* < 0.01, *** *p* < 0.001, and **** *p* < 0.0001) is shown.

Intrathecal and serum levels of anti-*A. muciniphila* IgG were measured in 61 patients with MS (Figure 2a). In order to characterize patients differing in anti-*A. muciniphila* IgG index, we formed two patient groups: one with low and one with high index, by selecting the twenty patients with the lowest and highest anti-*A. muciniphila* IgG index. With 100% of sensitivity and 100% of specificity, the cut-off was >0.05717. Twenty-one patients between 0.044 and 0.036 were excluded from the analysis as we took the top twenty low patients (0.005–0.0043) and the top twenty high-index patients (0.063–0.24). We verified that patients with prior treatments were not different from untreated patients. There were no significant differences between anti-*A. muciniphila* IgG indices in patients who had never been treated and those with prior treatment (Figure 2b). Therefore, the duration of stopping the treatments is long enough not to disrupt the dosages.

### 3.2. CSF Measures in Patients with MS with Different Anti-A. muciniphila IgG Index

Patients with low anti-*A. muciniphila* IgG index showed significantly higher QAlb as well as QAlb-QNorm values, suggesting altered BBB permeability, but not higher numbers of CSF-infiltrating cells (Figure 2c). Intrathecal IgG and IgM indices and synthesis were increased in patients with high anti-*A. muciniphila* IgG index (Figure 2c).

#### 3.2.1. B Cells in Patients with MS with Different Anti-*A. muciniphila* IgG Index

**Figure 3:**
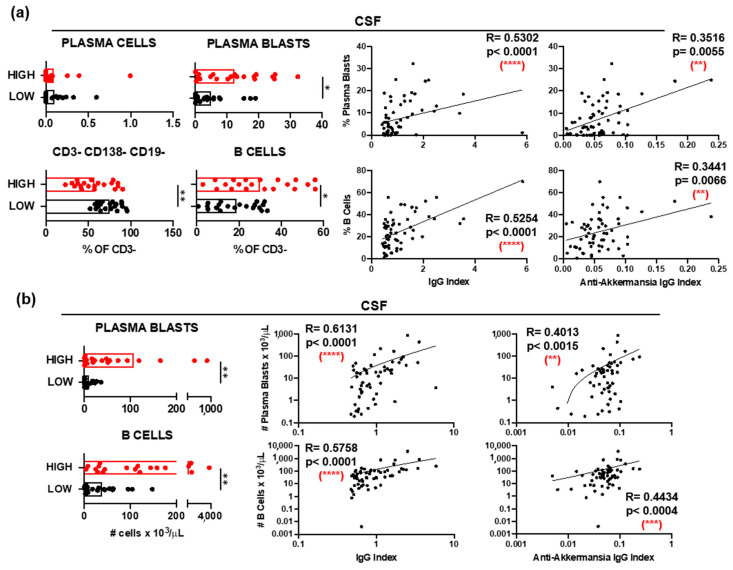

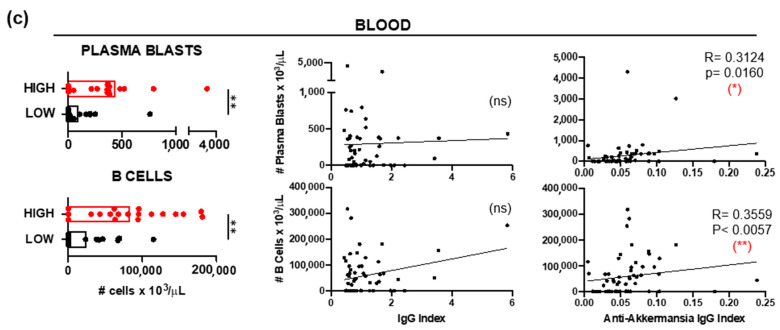
B cells and anti-*A. muciniphila* IgG indices. (**a**) Comparison between patients with high (red) and low (black) anti-*A. muciniphila* IgG indices of frequencies among CSF-infiltrating CD3− cells of plasma cells (CD138+ CD19−), plasma blasts (CD138+ CD19+), B cells (CD138− CD19+), and CD138− CD19− cells. Correlation between IgG indices (total- and anti-*A. muciniphila*) and frequencies of CSF-infiltrating plasmablasts and B cells. (**b**,**c**) Comparison between patients with high (red) and low (black) anti-*A. muciniphila* IgG indices of absolute numbers of CSF-infiltrating (**b**) and circulating (**c**) plasma blasts and B cells, as well as correlations of these numbers with IgG indices (total- and anti-*A. muciniphila*) (**b**,**c**). Each dot in the graphs corresponds to a single patient, and the bars show the mean. The Mann–Whitney test was used to compare two groups of patients. Linear correlation between variables was tested using Spearman’s r correlation. Statistical significance (* *p* < 0.05, ** *p* < 0.01, *** *p* < 0.001 and **** *p* < 0.0001) is shown. ns = not significant.

Immunophenotyping of CSF samples demonstrated significantly elevated relative frequencies (Figure 3a) and absolute numbers (Figure 3b) of plasma blasts (CD3− CD138+ CD19+) and B cells (CD3− CD138− CD19+) in patients with high anti-*A. muciniphila* IgG index. Further, these frequencies (Figure 3a) and numbers (Figure 3b) correlated positively with the total- and anti-*A. muciniphila* IgG indices. The absolute numbers of circulating plasma blasts and B cells were also significantly higher in patients with high anti-*A. muciniphila* IgG index (Figure 3c). While these counts did not correlate with the total IgG indices, they interestingly did with the anti-*A. muciniphila* IgG indices (Figure 3c).

#### 3.2.2. Th2 Cells in Patients with MS with Different Anti-*A. muciniphila* IgG Index

**Figure 4:**
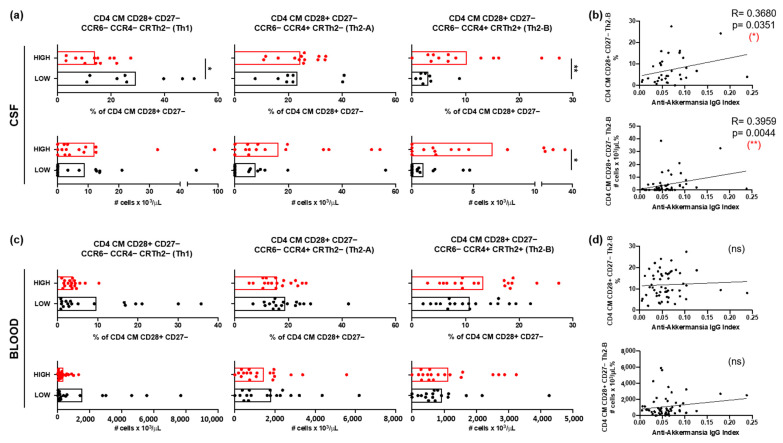
CSF-infiltrating T cells and anti-*A. muciniphila* IgG indices. (**a**,**c**) Relative frequencies and absolute numbers of CSF infiltrating (**a**) and circulating (**c**) CD4 CM CD28+ CD27− T cells with the following functional phenotypes Th1 (CCR6− CCR4− CRTh2−), Th2-A (CCR6− CCR4+ CRTh2−) and Th2-B (CCR6− CCR4+ CRTh2+) in patients with high (red) and low (black) anti-*A. muciniphila* IgG indices. (**b**,**d**) Correlation between IgG indices (total- and anti-*A. muciniphila*) and relative frequencies and absolute numbers of CSF-infiltrating (**b**) and circulating (**d**) CD4 CM CD28+ CD27− Th2 cells. Each dot in the graphs corresponds to a single patient, and the bars show the mean. The Mann–Whitney test was used to compare two groups of patients. Linear correlation between variables was tested using Spearman’s r correlation. Statistical significance (* *p* < 0.05, ** *p* < 0.01) is shown. ns = non significant.

The relative frequencies and the absolute numbers of CD4+ central memory (CM, CCR7+ CD45RA−) T cells expressing the coreceptor CD28 but not CD27, and with a Th2-B (CCR6− CCR4+ CRTh2+) functional phenotype were significantly higher in the CSF of patients with high anti-*A. muciniphila* IgG index (Figure 4a). Furthermore, both the relative frequencies and absolute numbers correlated with the anti-*A. muciniphila* IgG indices (Figure 4b). In contrast, the relative frequencies and absolute numbers of these cells in peripheral blood did not show differences between patients, nor did they correlate with anti-*A. muciniphila* IgG indices (Figure 4c,d).

**Figure 5:**
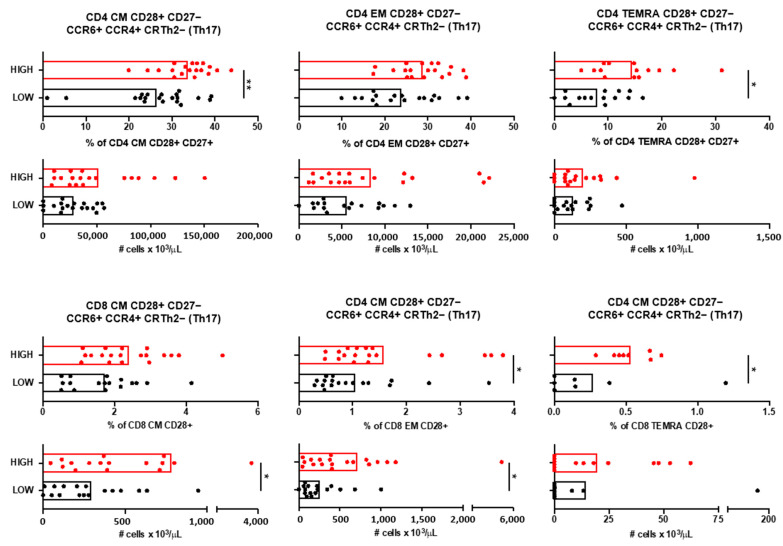
Circulating T cells and anti-*A. muciniphila* IgG indices. Relative frequencies and absolute numbers of circulating CD4 CD28+ CD27− and CD8 CD28+ T cells that have CM, EM, and TEMRA differentiation state and a Th17 (CCR6+ CCR4+ CRTh2−) functional phenotype in patients with high (red) and low (black) anti-*A. muciniphila* IgG indices. Each dot in the graphs corresponds to a single patient, and the bars show the mean. The Mann–Whitney test was used to compare two groups of patients. Statistical significance (* *p* < 0.05, ** *p* < 0.01) is shown.

Immunophenotyping of blood samples revealed that only T cells with a Th17 (CCR6+ CCR4+ CRTh2−) functional phenotype were increased in patients with high anti-*A. muciniphila* IgG indices (Figure 5). Circulating CM and TEMRA CD4+ T expressing the co-stimulatory molecules CD28 and CD27, as well as EM and TEMRA CD8+ T cells expressing the co-stimulatory molecule CD28 and all with a Th17 functional phenotype, were more frequent and/or more abundant in blood from patients with high anti-*A. muciniphila* IgG indices (Figure 5).

### 3.3. Characterization of Patients with Different Anti-A. muciniphila IgG Index

Demographic and clinical features did not differ between patients with low and high anti-*A. muciniphila* IgG index (Table 1).

**Figure 6:**
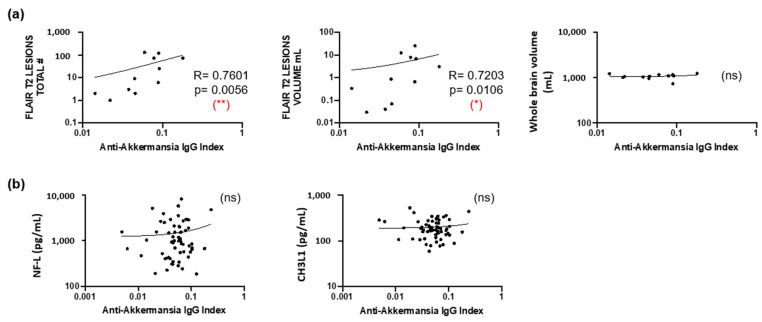
Association of anti-*A. muciniphila* IgG indices with MRI features. (**a**) Correlation between anti-*A. muciniphila* IgG indices and total number and volume of FLAIR T2 lesions and whole brain volume. (**b**) Correlation between anti-*A. muciniphila* IgG indices and intrathecal NF-L, CHI3L1, and number of monocytes in whole blood. Each dot in the graphs corresponds to a single patient, and the bars show the mean. Linear correlation between variables was tested using Spearman’s r correlation. Statistical significance (* *p* < 0.05, ** *p* < 0.01) is shown. ns = non significant.

Although brain MRI scans that have been obtained at the time of lumbar puncture were only available from twelve patients, these showed a statistically significant correlation between anti-*A. muciniphila* IgG indices and the total number and volume of FLAIR T2 lesions, but not with the whole brain volume (Figure 6a). We also addressed CNS damage and inflammation by using neurofilament light chain (NF-L) [26] and chitinase 3-like 1 (CHI3L1) [27] as biomarkers. Neither intrathecal NF-L nor CHI3L1 correlated with anti-*A. muciniphila* IgG indices (Figure 6b).

**Figure 7:**
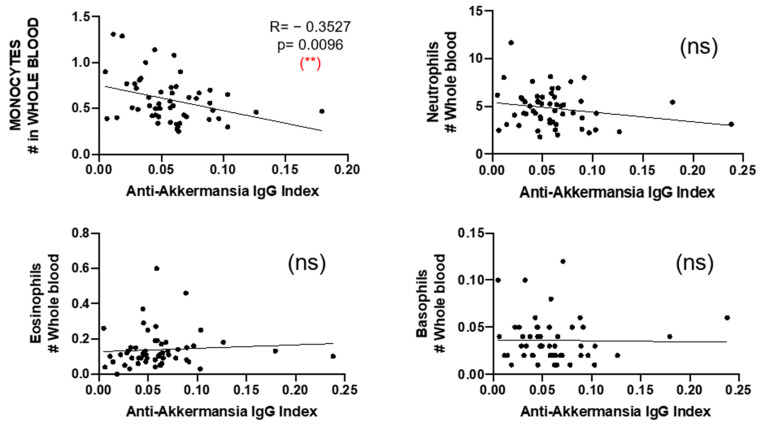
Correlation between anti-*A. muciniphila* IgG indices and number of neutrophils, eosinophils, and basophils in whole blood. Each dot in the graphs corresponds to a single patient, and the bars show the mean. The Kruskal–Wallis test was used to compare more than two groups of patients, and the Mann–Whitney test was used to compare two groups. Linear correlation between variables was tested using Spearman’s r correlation. Statistical significance (** *p* < 0.01) is shown. ns = non significant.

Finally, we compared the number of circulating neutrophils, eosinophils, basophils, and monocytes and found a negative correlation between the number of circulating monocytes and the anti-*A. muciniphila* IgG indices (Figure 7).

## 4. Discussion

In this study, we aimed to find new evidence supporting a role for intrathecal anti-*A. muciniphila* IgG in MS pathogenesis and thoroughly characterized patients with different anti-*A. muciniphila* IgG index. The comparison of patients with low and high intrathecal IgG production against this gut bacterium identified significant differences regarding BBB permeability, CSF infiltrates, pro-inflammatory circulating immune cells, as well as imaging features that indicate the role of these antibodies in MS pathogenesis.

Patients with MS with high anti-*A. muciniphila* IgG index also produced higher intrathecal IgG against other gut bacteria such as *P. melaninogenica*, *E. coli*, and *B. fragilis* [20,28], suggesting a leaky gut and a general translocation of gut commensal bacteria in these patients. However, intrathecal production of anti-*A. muciniphila* IgG did not correlate with serum levels of IgG specific for gut bacteria, which may indicate a selective recruitment into the CNS compartment of B cells producing these antibodies. The intrathecal synthesis of anti-*A. muciniphila* IgG is supported by higher intrathecal IgG and IgM synthesis in patients with high anti-*A. muciniphila* IgG index. The proliferation and activation of the corresponding secreting B cells present in the CNS and not in the periphery are probably favored by the inflammatory environment and interactions with immune cells. Indeed, there are higher amounts of CSF-infiltrating cells, which may be involved in antibody production, such as plasma blasts, B cells, and CD4+ CM CD28+ CD27− Th2-B cells. Furthermore, both the relative frequencies and absolute numbers of these cells correlated with anti-*A. muciniphila* IgG indices. CD4+ CM CD28+ CD27− Th2-B cells are probably relevant for providing B cell help since the downregulation of CD27 indicates repetitive stimulation with antigen and the expression of CCR4 and CRTh2 [24], a Th2 phenotype. Unexpectedly, patients with MS with high anti-*A. muciniphila* IgG index had lower QAlb, suggesting that trafficking of albumin and cells through the BBB uses different mechanisms. Indeed, passage of immune cells is considered an active phenomenon due to inflammation, while albumin could be considered as a passive transfer after BBB breakdown. The study of specific inflammatory mediators in correlation with the *A. muciniphila* IgG index would be informative. Indeed, a meta-analysis on 226 studies, including more than 13,000 patients with MS, clearly showed that TNF-α, CXCL8, CXCL13, IL-15, and IL12-p40 are increased in the CSF of MS compared to controls [29]. So, this cytokine/chemokine environment in the CNS is conducive to the attraction and proliferation of immune cells. In line with this general CNS inflammatory environment, we found inverse correlations between anti-*A. muciniphila* IgG index in CSF and granulocyte/monocyte number in blood, suggesting a recruitment of those cells in the CNS and a corresponding decrease in blood.

Patients with high anti-*A. muciniphila* IgG index also showed slightly higher pro-inflammatory Th17 cells. This is of interest since Th17 cells have been associated with many autoimmune diseases and are crucial in immune responses against bacterial infections [30] and also against bacterial translocation [31]. In the EAE model, gut microbiota dysbiosis was associated with an aberrant Th17 response in the gut, leading to exacerbation of neuroinflammation [6,7].

Despite the low number of patients from whom brain MRI scans were available at the time of lumbar puncture, anti-*A. muciniphila* IgG indices nicely correlated with the number and volume of FLAIR T2 lesions in the brain, suggesting a possible involvement of these antibodies in the demyelination process due to inflammation. The whole brain volume and markers of CNS damage/inflammation, such as NF-L [26] and CHI3L1 [27], did not correlate with anti-*A. muciniphila* IgG indices, which render an involvement of these antibodies in neurodegeneration unlikely.

In this study, we highlighted that IgG responses against *Akkermansia muciniphila* in CSF of patients with MS correlated with the presence of immune cells in CSF, activity of disease, and markers of CNS damage. However, in the literature, *Akkermansia muciniphila* is considered a probiotic for metabolic disorders and inflammatory bowel disease [31]. Gut *Akkermansia muciniphila* increase was beneficial in an animal model of neuroinflammation, increasing regulatory T cell responses [32], but depending on the composition of gut microbiota (environmental community), *Akkermansia muciniphila* exhibited variable effects on EAE course [33]. A better comprehension of *Akkermansia muciniphila* functions and interactions with other microbes and immune cells is crucial to develop new therapeutic options for multiple sclerosis.

## 5. Limitations

The design of this study was a cross-sectional study without the inclusion of healthy controls. The number of patients with MS was relatively small (*n* = 20 per group) due to the exclusion of 21 patients for anti-*Akkermansia muciniphila* IgG index cutting selection. Moreover, MRI data were available for only 12 patients. Further prospective studies on larger patient cohorts are needed to confirm the findings.

## 6. Conclusions

Our results demonstrate an association between intrathecal anti-*A. muciniphila* IgG and CSF-infiltrating cells that are known to be involved in antibody production, consistent with an intrathecal synthesis of anti-gut microbiota antibodies and a selective recruitment of specific immune cells into the CNS. The significant differences between patients with low and high anti-*A. muciniphila* IgG index regarding BBB permeability, MRI lesion load, or peripheral inflammation, while preliminary, suggests an involvement of these antibodies in MS pathogenesis.

## Figures and Tables

**Table 1:** Demographic and clinical features.

	All	Anti-*A. mucniniphila* IgG Index Low	Anti-*A. mucniniphila* IgG Index High	*p* ^1^
Number of patients	61	20	20	
Female/male ratio	2.05	1.2	3	0.32
Age at CSF puncture (years, mean ± sd)	36.2 ± 10.1	36.9 ± 11.6	35.8 ± 9.3	0.89
Age at disease onset (years, mean ± sd)	33.7 ± 9.2	35.0 ± 10.8	32.2 ± 9.4	0.54
Disease duration (Months, mean ± sd)	31.9 ± 58.8	29.9 ± 60.1	42.4 ± 72.9	0.89
RIS/CIS (%)	19.6	30.0	10.0	0.23
RRMS (%)	73.7	60.0	85.0	0.15
PMS (%)	6.5	10.0	5.0	0.90
CSF OCB Type II (%)	81.9	70.0	100.0	0.02
*HLA DR15* (%)	44.2	25.0	55.0	0.10
QAlb-QNorm > 0 (%)	19.6	35.0	5.0	0.04

^1^ Comparisons were performed using U-test (Mann–Whitney) and associations using Fisher’s Exact Test. RIS=Radio Isolated Syndrome. CSF = cerebrospinal fluid. OCB = Oligoclonal band. CIS = Clinically Isolated Syndrome. RRMS = Relapsing-Remitting MS. PMS = Progressive MS. sd = standard deviation. Q = Quotients. QAlb = Albumin concentration in CSF [mg/L]/Alb concentration in serum [g/L]. QNorm = [age/15] + 4 × 10^−3^).

## Data Availability

Data are available upon reasonable requests.
